# Automated assessment of steatosis in murine fatty liver

**DOI:** 10.1371/journal.pone.0197242

**Published:** 2018-05-10

**Authors:** Deepak Sethunath, Siripriya Morusu, Mihran Tuceryan, Oscar W. Cummings, Hao Zhang, Xiao-Ming Yin, Scott Vanderbeck, Naga Chalasani, Samer Gawrieh

**Affiliations:** 1 Department of Computer and Information Science, Indiana University Purdue University-Indianapolis, Indiana, United States of America; 2 Department of Pathology and Laboratory Medicine, Indiana University School of Medicine, Indianapolis, Indiana, United States of America; 3 University of Wisconsin-Milwaukee Research Foundation, Milwaukee, Wisconsin, United States of America; 4 Division of Gastroenterology and Hepatology, Indiana University School of Medicine, Indianapolis, Indiana, United States of America; Medizinische Fakultat der RWTH Aachen, GERMANY

## Abstract

Although mice are commonly used to study different aspects of fatty liver disease, currently there are no validated fully automated methods to assess steatosis in mice. Accurate detection of macro- and microsteatosis in murine models of fatty liver disease is important in studying disease pathogenesis and detecting potential hepatotoxic signature during drug development. Further, precise quantification of macrosteatosis is essential for quantifying effects of therapies. Here, we develop and validate the performance of automated classifiers built using image processing and machine learning methods for detection of macro- and microsteatosis in murine fatty liver disease and study the correlation of automated quantification of macrosteatosis with expert pathologist’s semi-quantitative grades. The analysis is performed on digital images of 27 Hematoxylin & Eosin stained murine liver biopsy samples. An expert liver pathologist scored the amount of macrosteatosis and also annotated macro- and microsteatosis lesions on the biopsy images using a web-application. Using these annotations, supervised machine learning and image processing techniques, we created classifiers to detect macro- and microsteatosis. For macrosteatosis prediction, the model’s precision, sensitivity and area under the receiver operator characteristic (AUROC) were 94.2%, 95%, 99.1% respectively. When correlated with pathologist’s semi-quantitative grade of steatosis, the model fits with a coefficient of determination value of 0.905. For microsteatosis prediction, the model has precision, sensitivity and AUROC of 79.2%, 77%, 78.1% respectively. Validation by the expert pathologist of classifier’s predictions made on unseen images of biopsy samples showed 100% and 63% accuracy for macro- and microsteatosis, respectively. This novel work demonstrates that fully automated assessment of steatosis is feasible in murine liver biopsies images. Our classifier has excellent sensitivity and accuracy for detection of macrosteatosis in murine fatty liver disease.

## Introduction

Increased fat accumulation in liver cells is the hallmark of non-alcoholic fatty liver disease (NAFLD), alcoholic fatty liver disease and hepatotoxicity from a wide range of medications [1].

Accurate identification and quantification of steatosis are important not only for making the diagnosis, but also for studying the pathogenesis of disease and determining the effects of therapeutic interventions [2, 3]. It is also essential for evaluating drug hepatotoxic signature in early drug development in animal models.

Current challenges in histological phenotyping of fatty liver, particularly in animal models, include limited availability of experienced liver pathologists, human variability in pathologists’ agreement on detecting and quantifying various histological features of liver disease, and the use of semi-quantitative manual grading scores that have limited range [4–9]. For example, semi-quantitative scores for macrosteatosis or lobular inflammation have limited grading bins (e.g., mild, moderate, severe or range from 0–3) and may not allow accurate assessment of changes in NAFLD lesions following therapeutic interventions; smaller changes may lead the pathologist to assignment of different grade if the changes fall on the border between grading bins, or keep the same grade if large changes fall within same grading bin.

Automation of the assessment of fatty liver disease histological lesions provides an innovative solution to these challenges [6, 10, 11]. It provides an automated, reproducible and continuous measurement of these features that permits accurate measurement of wide ranges in quantitative changes in these lesions. Using machine learning techniques, we previously developed a fully automated classifier capable of detecting and quantifying macrosteatosis in digital images of human liver biopsies [10].

However, fully automated methods to accurately assess steatosis in murine models is lacking.

Macrosteatosis is the cardinal lesion of NAFLD, and is commonly used as a major endpoint in therapeutic clinical trials in human NAFLD [12, 13]. Furthermore, development or improvement of macrosteatosis may reflect hepatotoxic or beneficial therapeutic signature in rodent models [14–17].

In this study, we develop automated classifiers for the identification of macro- and microsteatosis in murine models of fatty liver. The classifier for murine macrosteatosis is built by modifying and introducing new features to the original classifier we developed to measure macrosteatosis in humans [10]. We study the performance of this classifier in quantitative assessment of macrosteatosis in different models of murine fatty liver and evaluate the correlation of this quantification with semi-quantitative grading given by an expert pathologist. As microsteatosis is assessed as present or absent without further grading [4], the novel classifier for microsteatosis is built to detect the presence or absence of microsteatosis. To assess the accuracy of the classifiers, macro- and microsteatosis lesions identified by the classifiers on previously unseen digital images are verified by an expert pathologist.

## Materials and methods

### Mice liver biopsies

**Fig 1:**
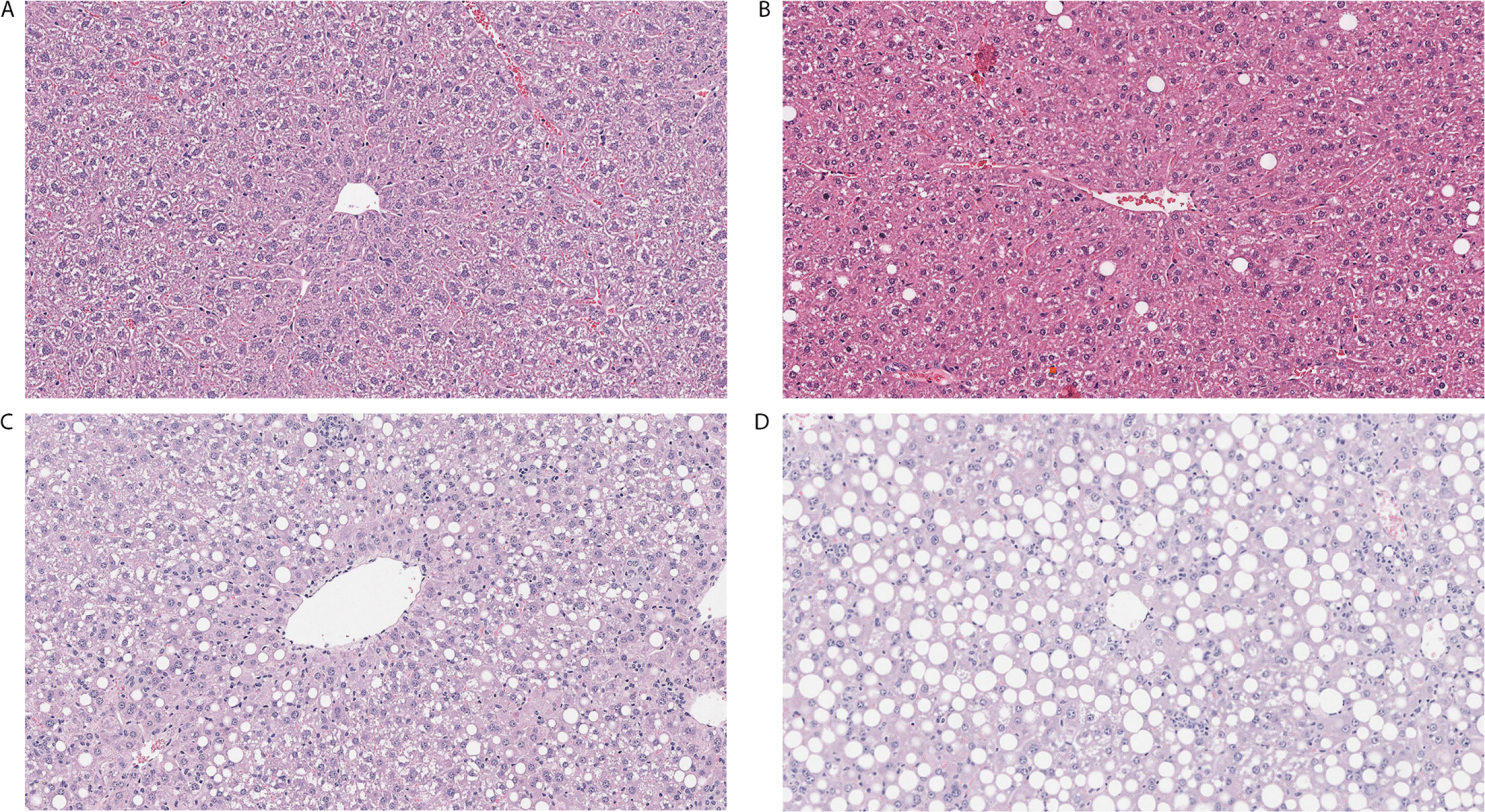
Representative Hematoxylin and Eosin images of different models used to induce fatty liver. A. Normal liver in wild type mouse fed chow diet. B. Mild fatty liver in wild type mouse fed high fat diet. C. Moderate fatty liver in GFP-LC3 mouse fed alcohol. D. Severe fatty liver in GFP-LC3 mouse fed high-fat high-carbohydrate diet.

The dataset used for this study consisted of digital images of 27 Hematoxylin and Eosin (H&E) stained slides of murine liver biopsies [9 with normal liver histology (no macrosteatosis: grade 0), 10 mild (macrosteatosis grade 1), 4 moderate (macrosteatosis grade 2), and 4 severe fatty liver (macrosteatosis grade 3)]. These mice were previously used in various experimental models to induce fatty liver. Fig 1 shows representative H&E stained sub-slides of each grade type.

All animal experimental protocols were approved by the Institutional Animal Care and Use Committee of Indiana University (IACUC). Animals were housed under approved conditions with 12 hour light dark cycle. C57BL/6 wild type and GFP-LC3[18] were bred in house. At 10 weeks of age, mice were placed on normal chow diet to produce normal liver, high fat diet (diet D12492, Research diets) to produce mild fatty liver or high-fat high-carbohydrate (HFHCD) diet, or chronic alcohol (29–36%) to produce moderate to severe fatty liver. For HFHCD diet, mice were given high fat diet along with the drinking water enriched with high-fructose corn syrup equivalent to a total of 42 g/L of carbohydrates. Drinking solution was made by mixing in drinking water at a ratio of 55% fructose (Acros Organics, Morris Plains, NJ) and 45% sucrose (Sigma- Aldrich, St. Louis, MO) by weight. Animals were provided ad libitum access to these diets for 10–24 weeks. Only liver biopsy slides from prior concluded studies were utilized for this study. Mice were first given Avertin (250 mg/kg, i.p.) for anesthesia, followed by cervical dislocation for euthanization. This procedure minimizes any suffering. All procedures are approved by the IACUC of Indiana University.

Liver biopsy H&E stained slides were studied and scored by an expert liver pathologist (OWC) according to NASH Clinical Research Network scoring system where steatosis grade range between 0–3 (0 < 5% steatosis, 1 = 5%-33%, 2 = 33%-66%, and 3 > 66%) [4].

Biopsy slides were scanned at 20X using Aperio Scan Scope CS system. The pathologist manually annotated the biopsy images using a custom built web-application to identify histological and anatomical features of the biopsy.

### Approach for macrosteatosis detection and quantification

**Fig 2:**
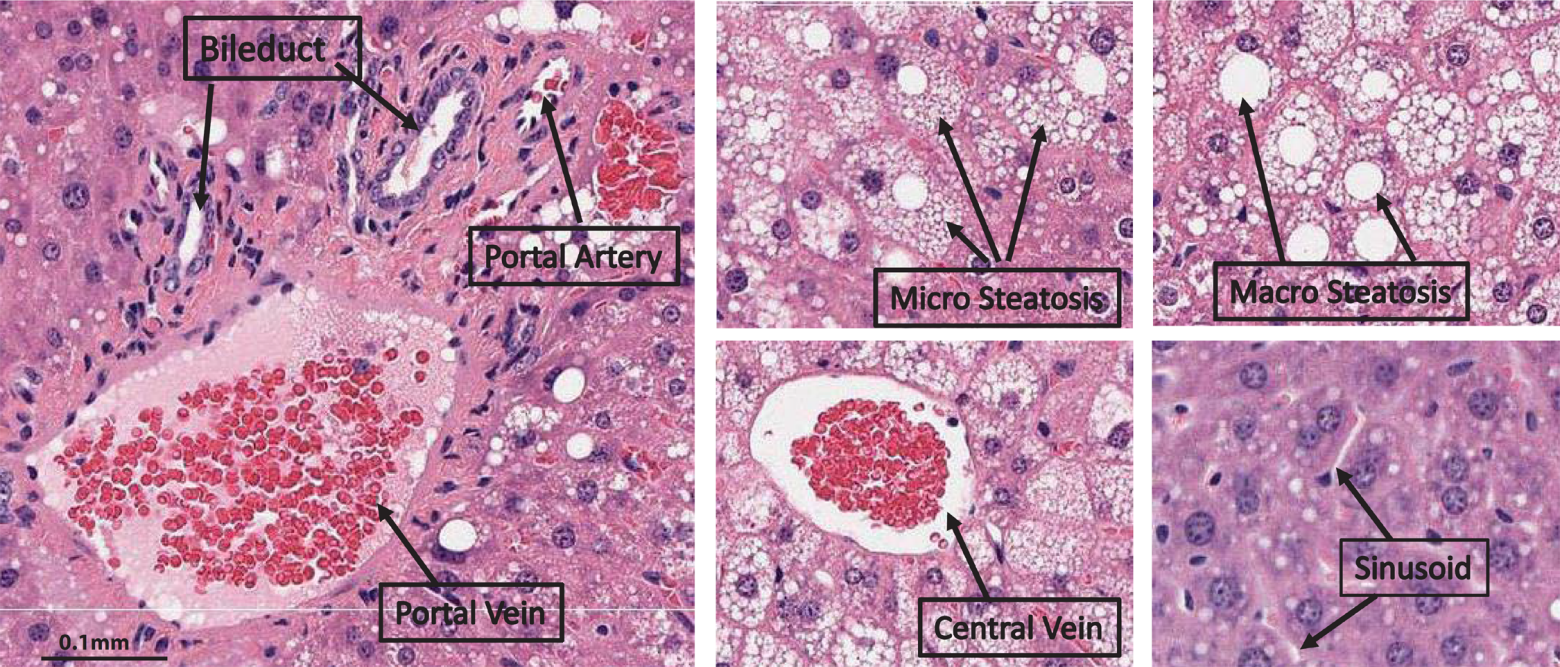
White regions in mouse liver biopsy.

A supervised machine learning model is trained using various regions which have a white center: macrovesicular steatosis, microvesicular steatosis, bile duct, portal artery, portal vein, and central vein. Fig 2 shows these regions.

Once the model identifies macrosteatosis regions among the other white regions, the percentage of macrosteatosis is calculated based on the area of tissue identified as fat. This value is then correlated with the semi-quantitative grade provided by the pathologist.

**Fig 3:**
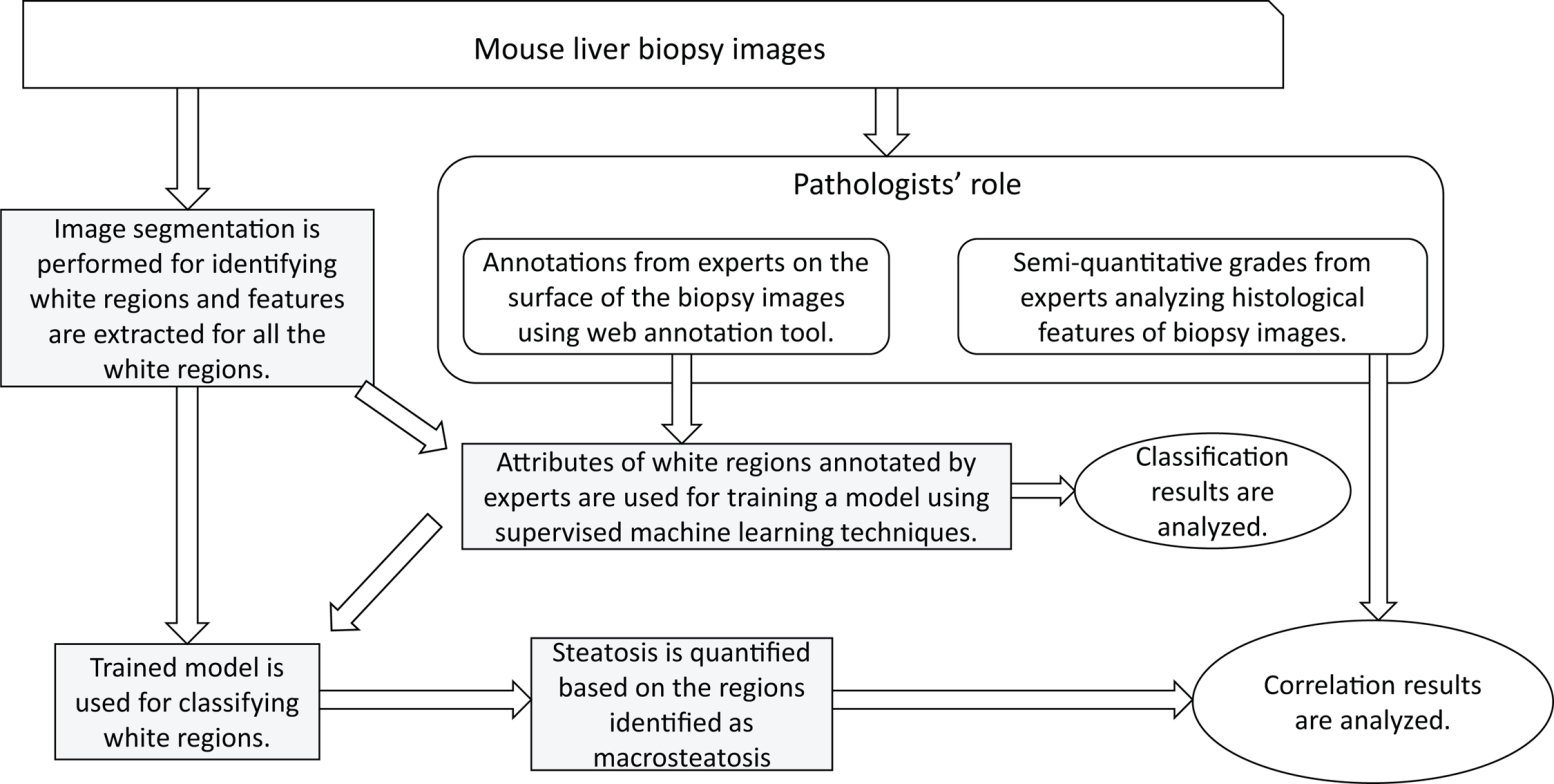
Overall flow diagram of the approach to automated macrosteatosis identification and quantification.

The processing steps in the identification and quantification of macrosteatosis are shown in Fig 3. More detailed explanation of each step is given below.

**Fig 4:**
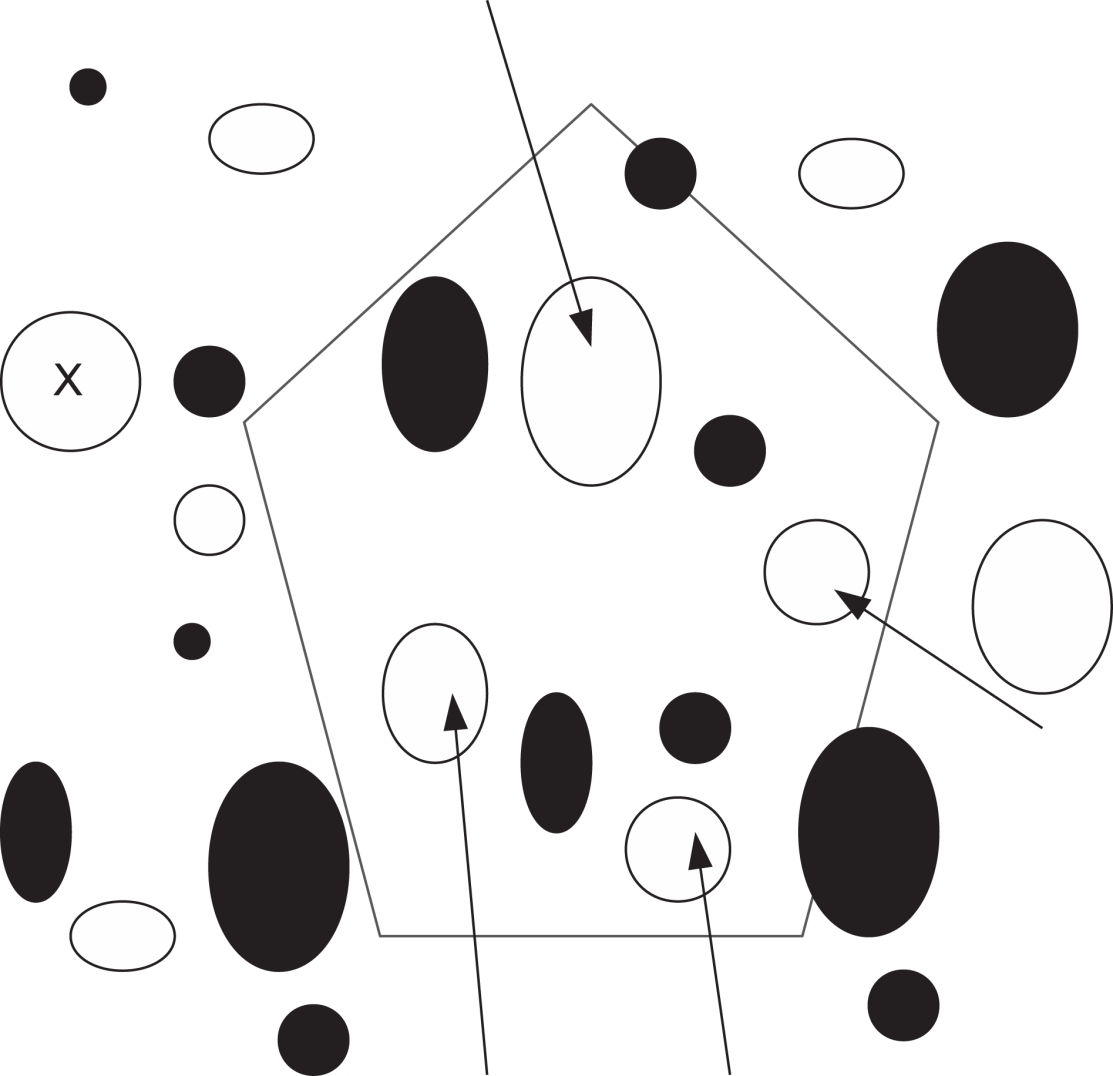
Graphic showing dark and white regions with point and polygon annotations. The white region labeled using point annotation (represented using 'X') is used for extracting attributes. A polygon annotation is shown using black polygon. The white regions falling within the polygon are indicated by arrows and these are the regions used for attribute extraction for the given label.

- **Obtaining the image**: The digital image from the Aperio Scan Scope CS system is stored in Aperio svs format. The resolution picked from svs format file is the 50% reduced one.
- **Identifying the tissue region**: A region growing algorithm is applied on the green channel of the 50% reduced RGB image to separate the tissue and background regions.
- **Identifying the white regions**: The intensity image obtained from green channel is filtered using a 3x3 averaging mask in order to smooth out noise. The intensity values in the image are then adjusted with a GAMMA correction value of 6 to make the image darker. *k*-means clustering is applied to the darkened image with a *k* value of 2 (i.e., 2-clusters are identified). Pixels in the image are now divided into two classes, white and black. Any white region bigger than 60 pixels is eliminated and the remaining white regions are used as starting points for further analysis.
- **Obtaining feature vector for each white region**: For each of the white regions obtained using the previous step, image features are computed to be used in learning the model. The details of the types of attributes extracted are further explained below in the Section on Feature vector representation.
- **Annotations**: The computed feature vectors of regions annotated by the pathologist are used as training patterns for learning the model. There are two types of annotations provided by the pathologist: points and polygonal regions. For point annotations, the individual white region on which the point is marked is considered. For polygonal annotations all the white regions falling within the given polygonal boundary annotation are considered and processed. Fig 4 shows a graphic with white and dark regions, point and polygon annotations. The white regions within a polygon pointed to by arrows in the figure are used for feature extraction for the label represented by the polygon. Similarly, the white region pointed to by point annotation is used for extracting attributes for the given label.
- **Learning the model:** Support vector machine (SVM) with a linear kernel is the machine learning technique used for learning the model for macrosteatosis identification from the expert pathologist’s annotations of several regions.
- **Macrosteatosis Identification:** Once the trained classifier model is ready, new (i.e., previously unseen) images are processed using the trained classifier: feature vectors are computed for all white regions and are used to classify each of the white regions, resulting in the identification of macrosteatosis.

### Approach for microsteatosis identification

As shown in Fig 2, microsteatosis could display different histological appearances on H&E stained liver biopsies, from very tiny cytoplasmic vesicles, to small cytoplasmic vesicles that do not displace the hepatocyte nucleus, to foamy appearance of the cytoplasm. A supervised machine learning model is trained using various regions: microvesicular fat, macrovesicular fat, lobular inflammation, portal inflammation, bile duct, portal artery, portal vein, central vein and sinusoid. This trained model is then used to identify the microsteatosis regions in the image among the other identified regions.

**Fig 5:**
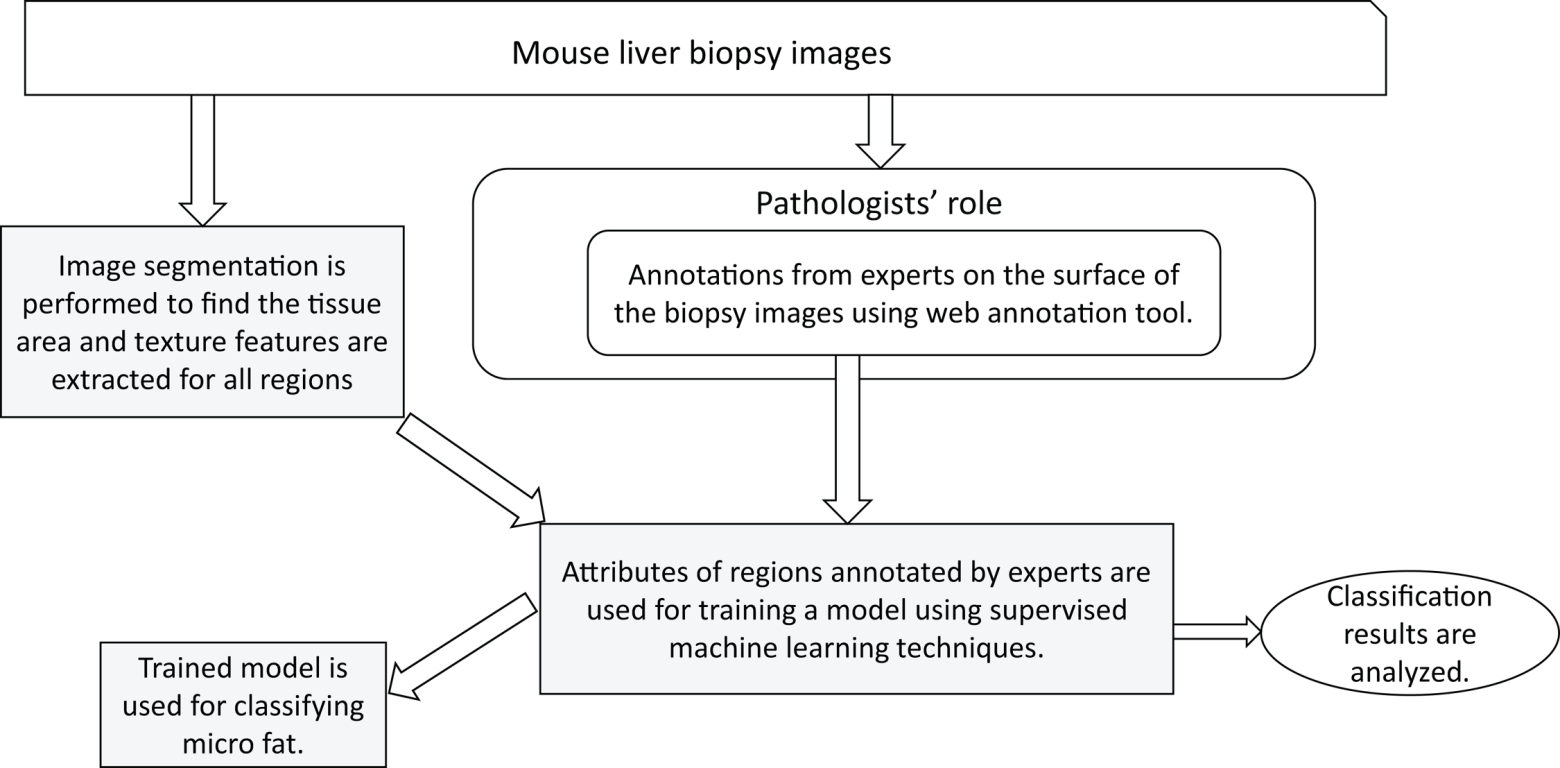
Overall flow diagram of the approach to automated microsteatosis identification.

The processing steps in the identification of microsteatosis are shown in Fig 5. More detailed explanation of each step is given below.

- **Obtaining the image**: The digital image from the Aperio Scan Scope CS system is stored in Aperio svs format. The resolution picked from svs format file is the 50% reduced one.
- **Identifying the tissue region**: A region growing algorithm is applied on the green channel of the 50% reduced RGB image to separate the tissue and background regions.
- **Obtaining feature vector for region (pixel wise)**: For each of the images generated after isolating the tissue area as described in step (b), Gabor filter based texture features are computed. The details of the feature extraction are further explained below in the Section on Feature vector representation.
- **Annotations**: The computed feature vectors of regions annotated by the pathologist are used as training patterns for learning the model. In the web-based annotation tool, the microsteatosis regions are denoted by the pathologist by drawing a polygon around the regions. The pixels falling within these annotated polygons are labeled as microsteatosis and the computed features for these pixels are used for training the model. Because the number of such pixels is too large, in order to reduce the computation time for the training phase, we randomly subsampled 0.02% of the pixels annotated as microsteatosis and 0.001% of the pixels annotated as any other histological feature.
- **Learning the model:** SVM with a linear kernel is the machine learning technique used for learning the model for microsteatosis identification from the expert pathologist’s annotations of several regions.
- **Microsteatosis identification:** Once the trained classifier model is ready, new (i.e., previously unseen) images are processed using the trained classifier: feature vectors based on Gabor texture features are computed for all regions which are then used to classify them as microsteatosis or non-microsteatosis regions.

### Feature vector representation

In our prior study [10], we used image texture statistics at various scales (σ = 0,1,2,4,8) as feature vectors to be used as input to the classifier. In this study, we have extended this feature set to improve the accuracy of identification. We added a new set of textural features using Gabor filters [19, 20] in detected white regions or in annotated macrosteatosis regions. For each of the white regions, the surrounding area of 20 pixels in all directions is considered for extracting these attributes. Also, a rectangular region of 25 pixels around the white region is considered for feature extraction.

Gabor filters, which are band pass filters at various frequencies and orientations, are used in this study for texture analysis. The impulse response of a Gabor filter is the product of a sinusoidal plane wave at a given frequency (f = 1/λ) and orientation (*θ*) with a Gaussian function at a given scale (*σ*).

The equation of Gabor function in spatial domain is given below [21–24]:

$$g\left(x,y;\lambda ,\theta ,\psi ,\sigma \right)=\exp\left(-\frac{x^{\text{'}2}+y^{\text{'}2}}{2\sigma^{2}}\right)\exp\left(i\left(2\pi \frac{x^{\prime }}{\lambda }+\psi \right)\right)$$

where X' = x cos θ + y sin θ and y' = –x sin θ + y cos θ, are the rotated coordinates to account for the filter orientation, λ = 1/f is the period of the sine wave, θ is the orientation of the Gabor filter, σ is the width of the Gaussian envelope, ψ is the phase offset of the sine wave, and i = √-1. Thus, the function g(x,y;λ,θ,ψ,σ) defines the convolution kernel. Filtering is performed in the frequency domain using fast Fourier transforms as it is computationally faster.

In the macrosteatosis identification, we have used four orientations (θ = 0°, 45°, 90°, and 135°) for the Gabor filters and for each orientation five radial frequencies which are one octave apart, for a total of 20 Gabor filters (4 orientations x 5 frequencies) [25]. These filters are applied to 7 images (3 for RGB channels separately, 3 for HSV channels separately and one for the gray level image) resulting in a total of 140 Gabor filter features.

These, combined with the features from our prior study [10], results in a total of 554 features which have been used in training the classifier.

In the microsteatosis identification, we have used four orientations (θ = 0°, 45°, and 135°) for the Gabor Filters and for each orientation ten radial frequencies which are one octave apart, for a total of 40 Gabor filters (4 orientation × 10 frequencies) [25]. These filters are applied to a single image (gray scale), resulting in 40 Gabor filter features.

### Macrosteatosis quantification

After macrosteatosis regions are identified by the trained classifier, the percent area of macrosteatosis in a biopsy image was quantified. Pathologists provide semi-quantitative macrosteatosis grade based on their perception of percentage macrosteatosis area. And hence, in this study, the ratio of measured macrosteatosis area to the total tissue area is calculated so that it can be compared with pathologist’s grades. For each of the white regions, the area is already calculated as part of computing the morphological features. The sum of areas of all white regions identified by the classifier is the “macrosteatosis area”. The tissue area is computed by using the background mask extracted in the white region identification process. The image area in the regions where mask is not present is considered as tissue and the space occupied by this region in pixels is the “tissue area.”

### External validation of classifier’s accuracy

To verify the accuracy of the classifiers identifications of lesions as macro- or microsteatosis, liver biopsy images previously unseen by the classifiers that have not been used in the training of the classifier were used in this step. For macrosteatosis validation, fifty randomly identified macrosteatosis lesions were selected and classified using the automated algorithm. These classification results were examined by the study pathologist to determine the validity of the identification.

For microsteatosis validation, one hundred and ten random sub slides from a pool of eleven previously unseen slides were examined by the study pathologist to determine the validity of the identification. We used a larger number of microsteatosis identified lesions for validation in order to properly represent the slides based on the significantly smaller size of these lesions compared to macrosteatosis lesions. The study pathologist assessed each identified (microsteatosis or not microsteatosis) lesion by the classifier as correct or incorrect. Accuracy was calculated as percent correctly identified with macro- or microsteatosis by the classifier as determined by the gold standard, the study expert pathologist.

## Results

### Training datasets for the classifiers

The training data for learning the macrosteatosis model was the white regions annotated by the expert, including 2256 macrosteatosis annotations from 11 liver biopsy images. The training data for learning the microsteatosis model was polygonal regions annotated as microsteatosis by the expert, including 570 microsteatosis annotations from 5 liver biopsy images.

### Classifiers’ performance metrics

Evaluation of accuracy of macro- and microsteatosis identification was performed using a 10-fold cross validation in the training set. For macrosteatosis prediction, the model’s precision, recall (sensitivity) and area under the receiver operator characteristic (AUROC) were 94.2%, 95%, 99.1%, respectively. The classifier’s accuracy was 84.2%.

**Fig 6:**
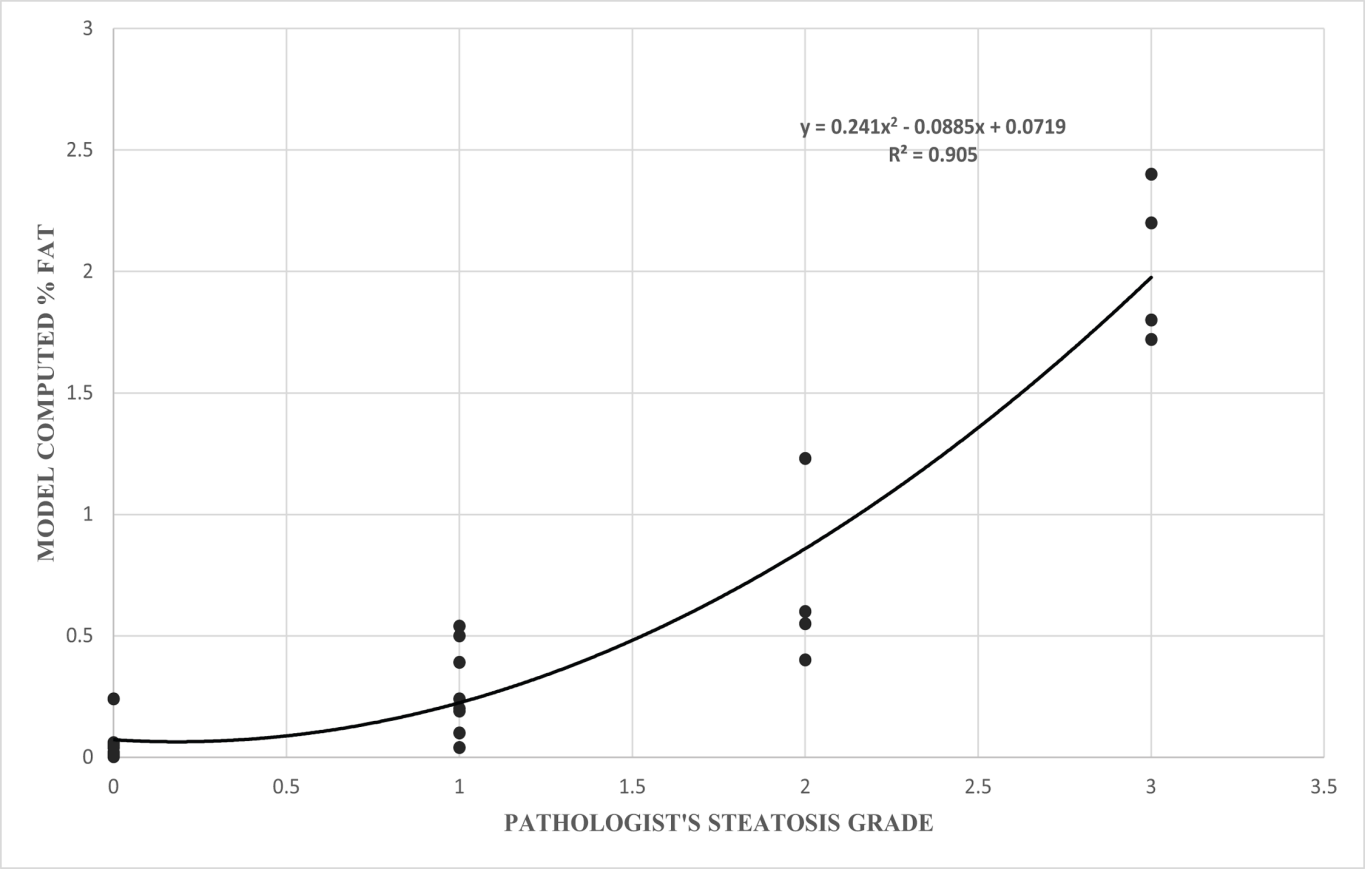
A scatter plot showing correlation between model-computed percentage steatosis for mouse biopsies with respect to the corresponding pathologist grade.

**Fig 7:**
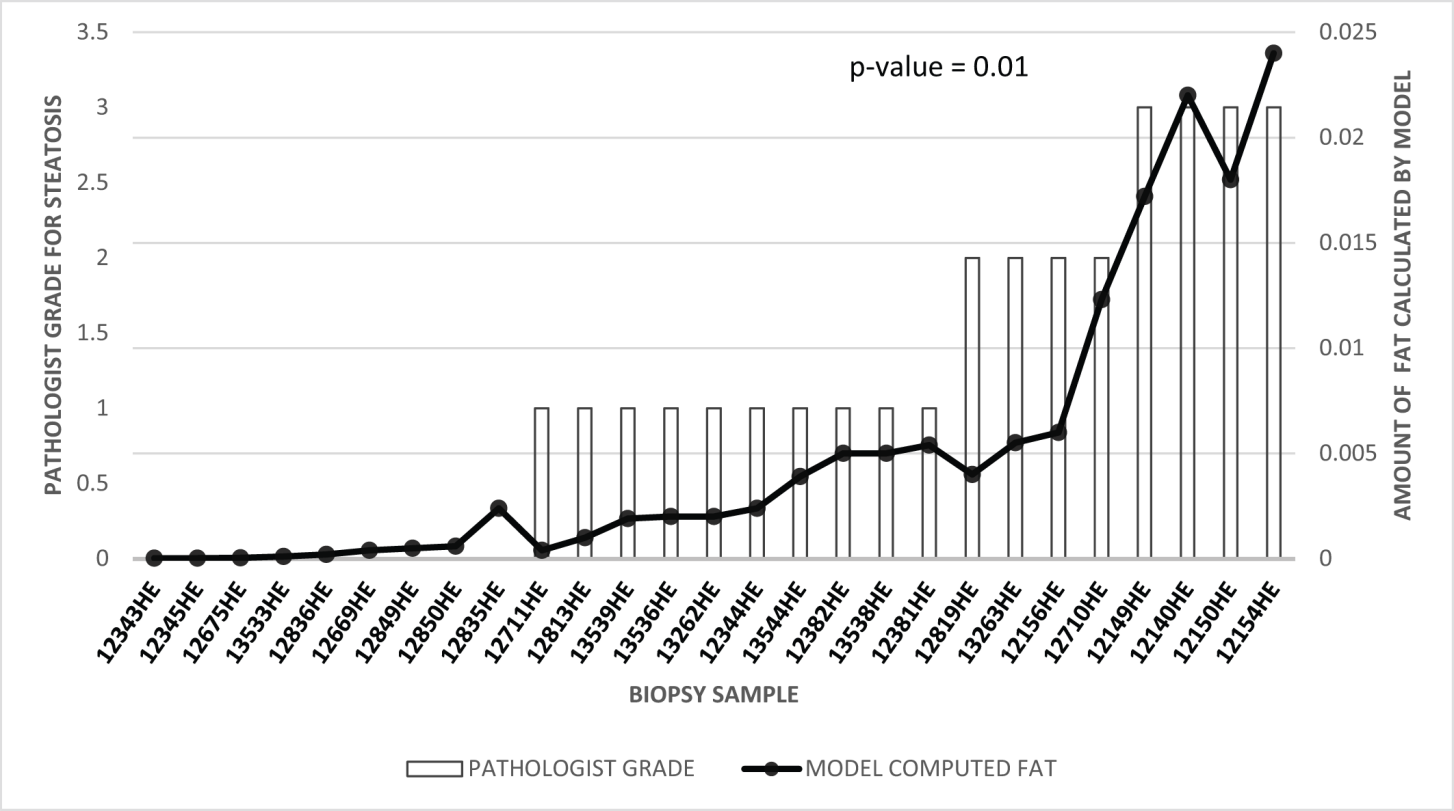
Mixed line/bar chart showing the correlation between the computed percentage steatosis with the expert pathologist grade. The pathologist grade for mouse samples is shown as a bar matching the left axis, and the computed percentage steatosis is shown as a line matching the right axis.

When correlated with pathologist’s semi-quantitative grade of macrosteatosis, the model fits with a coefficient of determination *R*^2^value of 0.905. Fig 6 shows the *2*^*nd*^ order polynomial curve fitted to the data points. The strong correlation between quantitative results obtained by the algorithm developed in this study for estimating the percentage macrosteatosis and the pathologist macrosteatosis grades are shown in Fig 7 (p = 0.01 after performing two-sample t-test with a degree of freedom of 26). Table 1 shows the confusion matrix of the model built for macrosteatosis prediction.

**Table 1:** The confusion matrix and metrics for the model built to detect macrosteatosis.

Actual	Predicted					
	*Central Vein*	*Macro Fat*	*Bile Duct*	*Portal Vein*	*Portal Artery*	PRECISION (%)
***Central Vein***	230	8	0	25	0	87.45
***Macro Fat***	6	343	0	12	0	**95.01**
***Bile Duct***	0	1	17	27	5	30.91
***Portal Vein***	15	8	14	298	2	87.9
***Portal Artery***	2	4	4	7	13	37.14
**RECALL (%)**	90.91	**94.23**	41.46	79.25	52	

For microsteatosis prediction, the model’s precision, recall and AUROC were 79.2%, 77%, 78.1%, respectively. The classifier’s accuracy was 78.7%. Table 2 shows the confusion matrix of the model built for microsteatosis prediction.

**Table 2:** The confusion matrix and metrics for the model built to detect microsteatosis.

Actual	Predicted		
	***Micro Fat***	***Others***	**PRECISION (%)**
***Micro Fat***	7109	1862	**79.24**
***Others***	2116	7660	78.35
**RECALL (%)**	**77.06**	80.445	

**Fig 8:**
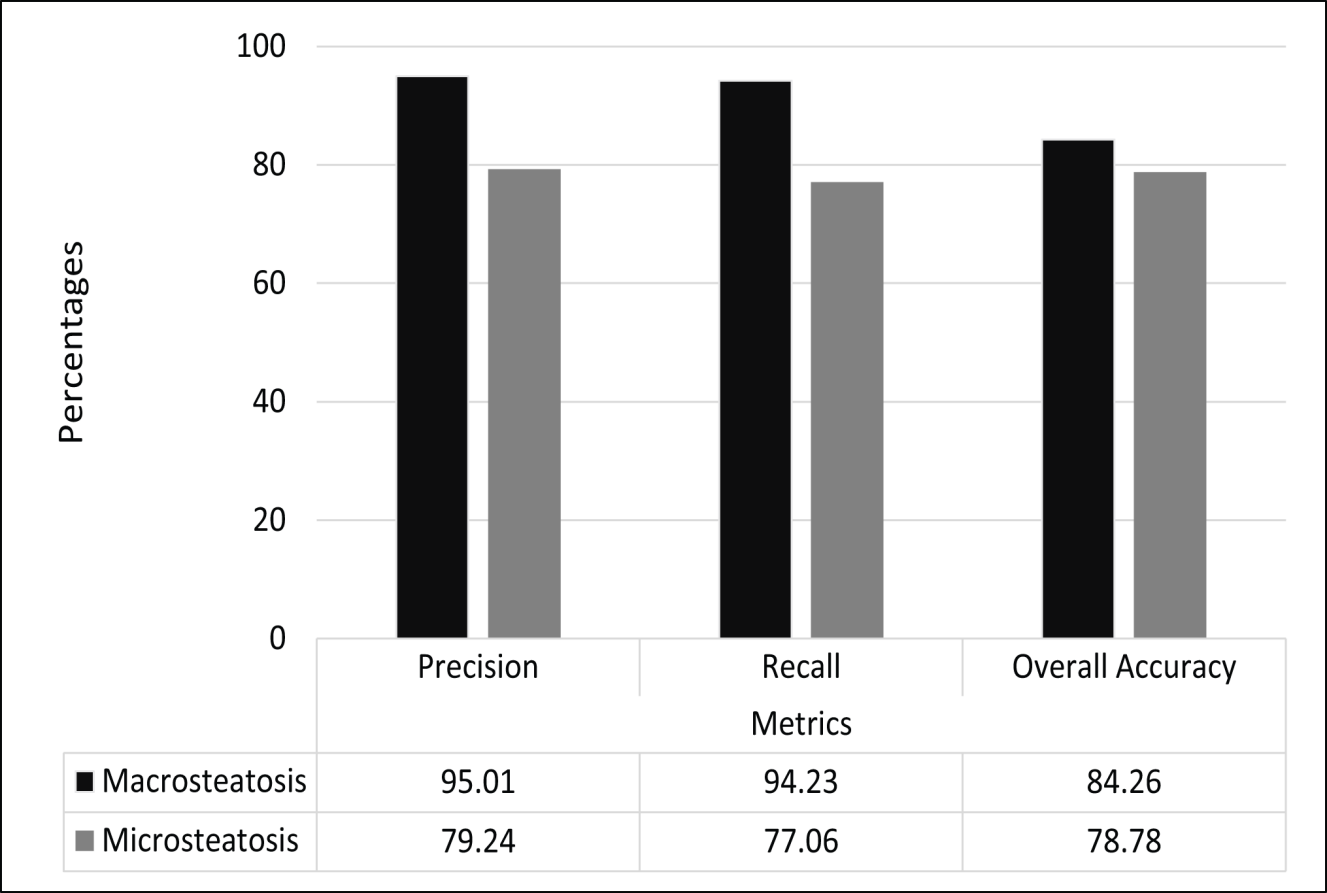
A graph which represents the different models generated and their respective metrics.

Fig 8 presents a visual summary of the performance metrics for the different steatosis identification models developed using supervised machine learning methods described above.

### External validation of the classifiers’ accuracy

**Fig 9:**
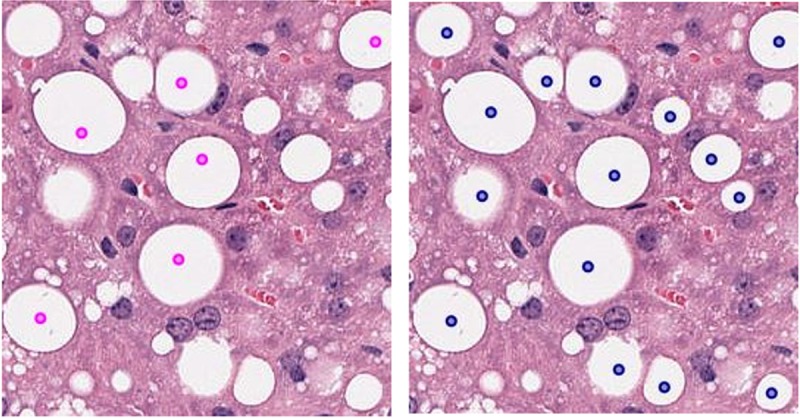
Comparison of annotated macrosteatosis lesions by pathologist v/s our model. The left slide contains annotations given by the pathologist (marked as a point in magenta). The right slide contains macro fat lesions detected by the model (marked as point in dark blue).

**Fig 10:**
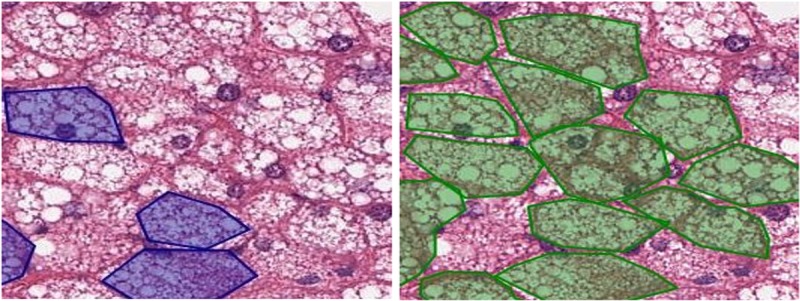
Comparison of annotated microsteatosis lesions by pathologist v/s our model. The left slide contains annotations given by the pathologist (marked as polygons in dark blue). The right slide contains micro fat lesions detected by the model (marked as polygons in green).

**Fig 11:**
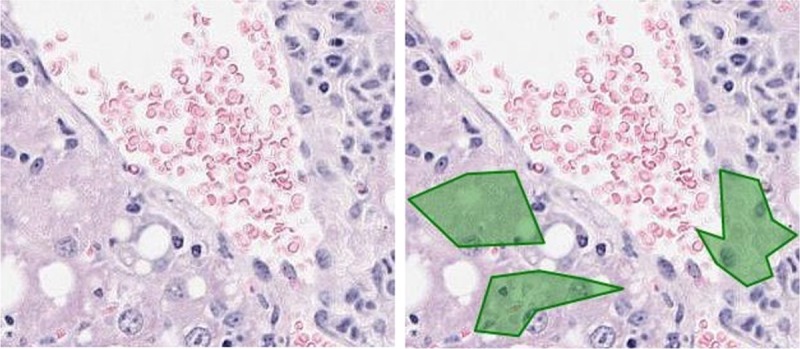
Some misclassified instances of microsteatosis lesions. The left slide contains no micro fat according to the pathologist. The right slide contains wrongly classified micro fat lesions detected by the model (marked as polygons in green).

The expert pathologist validated classification results on unseen slides for detection of steatosis. For macrosteatosis, evaluation by the study expert pathologist of the accuracy of predictions made on biopsy samples images unseen previously by the classifier, showed 100% accuracy. For microsteatosis, of the one hundred and ten random sub slides the detection accuracy was 63%. The pathologist also confirmed that for every unseen slide, the classifier had accurately detected microsteatosis on at least one sub slide (i.e., 100% sensitivity for detecting ***any*** microsteatosis). It is practically impossible for the pathologist to label the massive number of macro- or microsteatosis lesions present on an individual slide, but the classifier can do that automatically. Here we show examples in Fig 9 (for macrosteatosis) and Fig 10 (for microsteatosis), where both the pathologist’s and classifier’s labels fell on the same lesion in the hepatocytes but the classifier correctly labeled many other lesions in the same area of the biopsy. Fig 11 shows an example of misclassified microsteatosis.

Other histological features of fatty liver such as lobular inflammation or ballooning, or artifacts on the slides did not affect the performance of the model for detection of macrosteatosis, as evidenced by the 100% accuracy of the classifier. However, they did for microsteatosis, where ballooning or artifact was confused by the classifier as microsteatosis in some mislabeled cases by the classifier (Fig 11).

## Discussion

Despite intense interest in automating the assessment of steatosis in humans and animal models, there is lack of robust fully automated methods to detect and quantify steatosis in mice. This study demonstrates that fully automated assessment of steatosis is feasible in murine liver biopsy images. Our classifier has excellent precision, sensitivity and accuracy for detection and quantification of macrosteatosis in mice with fatty liver disease regardless of the model used to induce fatty liver. We also developed a novel model for identification of microsteatosis that has good precision and high sensitivity in murine models of fatty liver.

We used a systematic approach to build and validate our models. Two sets of liver biopsies images were used. The first set, for building, training and internally validating the models. The second set of unseen liver images was used to externally validate the performance of the classifiers. Supervised machine learning approaches were used to remedy the problem of inter- and intra-observer error in semi-quantitative assessment of liver histological lesions. We used biopsy samples which are in different severity of macrosteatosis as well as controls with no macro- or microsteatosis.

There are several prior efforts, including from our group, to automate macrosteatosis assessment in humans [10, 26]. However, robust validated methods for automated assessment of steatosis in animal models of fatty liver are lacking. Others have also attempted quantifying macrosteatosis using machine learning and digital image analysis in rats and mice [27–29]. In one study for example, automated detection of fat droplets in rats showed more than 90% specificity and sensitivity [28], but the study lacked external validation of the identified steatosis lesions. In rats fed methionine-choline-deficient plus high-fat diet, the same group noted no correlation between their automated method of quantifying steatosis versus pathologist’s visual estimate for mild and moderate degrees of steatosis. Our method for quantifying macrosteatosis has excellent correlation with pathologist’s semi-quantitative assessment with a *R*^2^ value of 0.905. The severity of macrosteatosis and its zonal distribution did not affect the classifier’s performance, which remained accurate across all severities of fatty liver slides included in the training and validation phases of the study and regardless of the zonality of macrosteatosis. All lesions detected by the classifiers on unseen images were validated as true by the study pathologist.

It should be noted that our study had no bias when biopsy samples were selected. Therefore, our algorithm is robust to variations in the intensity if H&E stain on the sample and to the noise introduced during the image acquisition and/or slide preparation process.

Macrosteatosis is the cardinal lesion of NAFLD and a key feature of alcoholic liver disease and drug hepatotoxicity [30, 31]. Therefore, accurate detection and quantification of this lesion is essential to studying these disease processes. While imaging modalities such as ultrasound, computed tomography, and magnetic resonance imaging, have been routinely used in clinical care of humans with fatty liver disease as non-invasive tools to assess hepatic steatosis, these methods are not suited for assessment of steatosis in murine models of fatty liver, where liver biopsy remains a viable and routinely used tool to study the pathogenesis of the disease or measure the effects of new therapeutic agents in preclinical animal studies. Current assessment of steatosis in liver biopsy relies on the availability and access to highly skilled pathologists who perform a manual evaluation of liver biopsy slides and generate a semi-quantitative score for macrosteatosis. This assessment is subject to human variability. Our fully automated method allows accurate, reproducible and continuous measurement of macrosteatosis. Further, the H&E stain is the most widely used in clinical and research practices to evaluate macrosteatosis. We therefore developed this method using only the H&E stains so that no additional special stains will be needed to make this measurement.

Microsteatosis is uncommonly seen in the setting of human non-alcoholic fatty liver disease, but when present may indicate increased disease severity [32]. It is also seen in the setting of drug induced hepatotoxicity [31]. It is therefore an important hepatic lesion to identify especially during drug development in animal models [14, 33]. To our knowledge, no prior attempts have been undertaken to automate the identification of microsteatosis in liver biopsy images. Although our results show promising performance of this novel classifier to detect microsteatosis, there is room for further improvement of the classifier’s accuracy for detection of this challenging lesion. Some of the classifier’s labels were deemed incorrect by the pathologist due to different reasons, such as the classifier confusing artifact on the slide as microsteatosis (Fig 11), or incorrectly labeling ballooning of the hepatocytes as microsteatosis. We believe these problems with microsteatosis detection can be improved by adding far more annotations by the pathologist of microsteatosis to train the model and by creating different types of features (orientation based).

In summary, we have developed and validated a fully automated and highly accurate classifier to detect and quantify macrosteatosis and also a novel model to detect microsteatosis in murine fatty liver. The ability to accurately assess macro- and microsteatosis on the H&E stained slides without additional sophisticated stains is one strength of this work. This classifier could be a valuable tool in assessing murine fatty liver disease in various experimental and therapeutic conditions.
